# How bacteria block their own biofilms

**DOI:** 10.1016/j.jbc.2021.100392

**Published:** 2021-03-04

**Authors:** Thomas Delerue, Kumaran S. Ramamurthi

**Affiliations:** Laboratory of Molecular Biology, National Cancer Institute, National Institutes of Health, Bethesda, Maryland, USA

**Keywords:** E. coli, Staphylococcus aureus, cystic fibrosis, secondary metabolite, natural products

## Abstract

Bacterial biofilms are surface-associated multicellular communities that are highly resistant to removal. Scheffler *et al.* discovered that *Pseudomonas aeruginosa* secretes a small molecule that inhibits other *P*. *aeruginosa* cells from adsorbing to surfaces by interfering with type IV pili dynamics. The inhibition of cell adsorption could present a method to prevent biofilm formation on sensitive surfaces in hospitals and industry.

Surface contamination by microbes in hospitals and in the food industry can threaten human life. Often, bacteria exist on these surfaces as resilient multicellular communities, termed “biofilms,” in which cells adhere to each other and the surface, embedded in an extracellular matrix (1). As such, surface-adsorbed cells in biofilms are difficult to remove for proper sterilization of sensitive equipment and environments (2). Biofilm assembly initiates with the surface adhesion of one or few cells. This adhesion is dependent on physical and chemical forces and can vary according to the environment, bacterial growth state, and properties of the surface itself. To enhance this initial attachment, some species have developed specialized appendages. For example, *P. aeruginosa*, a bacterial opportunistic human pathogen that is a frequent cause of hospital-acquired infections, elaborates “Type IV” pili, an extracellular polymer that the cell can actively extend and retract to sense surfaces and adhere to them (3). Recently, it was reported that *P. aeruginosa* induces expression of virulence factors upon initial attachment, making this initial adherence step a potential target for the development of new antimicrobial drugs (4).

Many bacteria, including *P. aeruginosa*, use secondary metabolites to affect their population dynamics, including triggering the formation of biofilms and initiating their subsequent dispersal, inducing the production of virulence factors, and interfering with the growth of other competing species. However, it was not clear whether bacterial molecules might also inhibit the first steps in biofilm formation. Scheffler *et al.* (5) therefore screened cell-free media from *P. aeruginosa* cultures to search for molecules that would disrupt *P. aeruginosa* early cell surface attachment and consequently promote cell dispersal in early-stage biofilms. To achieve this, the authors first developed a high-throughput microscopy-based adsorption assay (which they termed “DISPEL”) to assess the dispersal activity of *P. aeruginosa* cell-free culture media harvested at different growth phases. They discovered that media harvested from stationary-phase bacterial cultures could robustly disperse surface-attached *P. aeruginosa*. The investigators purified a molecule responsible for this dispersal activity and, by employing mass spectrometry and NMR, identified it as 2-methyl-4-hydroxyquinoline (MHQ): a result that was consistent with their later observation that commercially available MHQ effectively dispersed surface-attached *P. aeruginosa*.

MHQ has previously been characterized as a secondary metabolite, but the origins and functions of this compound have not been investigated. The structural similarity of MHQ with other alkyl quinolones implicated in quorum sensing, such as 2-heptyl-4-hydroxyquinoline (HHQ) and 2-heptyl-3,4-dihydroxyquinoline (PQS), led the authors to consider that the *pqsABCDE* operon, responsible for quinolone synthesis, could be responsible for synthesis of MHQ as well (6). Accordingly, analysis of culture media harvested from a *P. aeruginosa* strain lacking this operon showed the absence of MHQ production, demonstrating that these genes are required for MHQ synthesis. Interestingly, despite the structural similarity of HHQ, PQS, and other alkyl quinolones of various tail lengths synthesized by this operon with MHQ, these other alkyl quinolones did not exhibit dispersal activity against surface-attached *P. aeruginosa*.

The authors next examined how MHQ inhibits early attachment. The addition of MHQ to *P. aeruginosa* did not kill the cells, nor did it disrupt the cells’ membrane integrity, ruling out some of the most standard antibiotic mechanisms. However, upon examination of MHQ-treated cells by light microscopy, the authors observed that the rod-shape *P. aeruginosa* cells that were initially adsorbed onto the surface *via* a cell pole (and therefore oriented vertically) switched to a horizontal orientation, presumably to increase cell surface contact in an effort to maintain adherence (Fig. 1). This phenotype was similar to that resulting from cells without the ability to elaborate type IV pili. Additionally, the authors observed that MHQ-treated cells displayed reduced twitching motility (7)—a bacterial surface translocation driven by type IV pili powered by cycles of polymerization, adhesion, and retraction—compared with wild-type cells. To directly visualize the effect of MHQ treatment on pilus dynamics, the authors fluorescently labeled pili and examined pilus activity using fluorescence microscopy, which revealed that pilus extension and retraction were diminished in the presence of MHQ.

**Figure 1:**
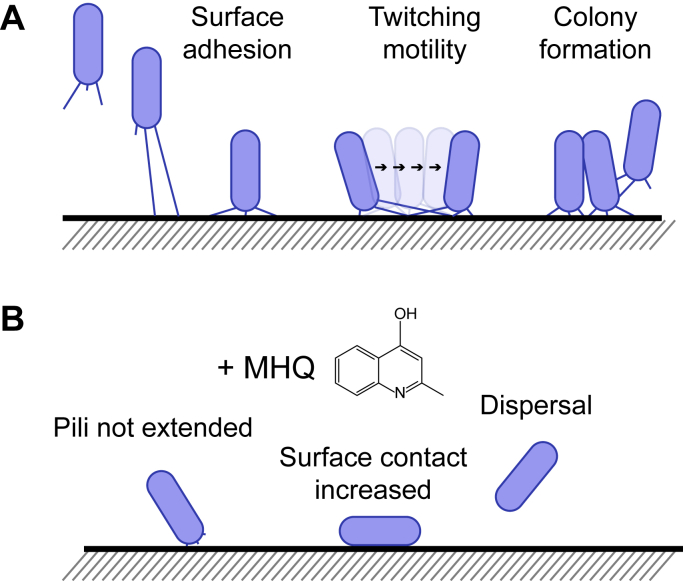
**Initial attachment of *P. aeruginosa* onto a surface is inhibited by the secreted molecule MHQ.***A*, *P. aeruginosa* cells adhere to a surface *via* type IV pili (*left*). *Center*, pilus retraction permits adsorbed cells to move *via* “twitching motility.” *Right*, ultimately, other cells adsorb to the surface and form a colony. *B*, in the presence of 2-methyl-4-hydroxyquinoline (MHQ), cells fail to display pilus dynamics (*left*). As a result, cells fail to adsorb properly, do not display twitching motility, and eventually disperse.

The authors proposed that preventative administration of purified MHQ may limit *P. aeruginosa* growth on sensitive surfaces. Moving forward, it will be interesting to test if the coadministration of MHQ with antibiotics, for example, would increase bactericidal activity *via* partial or complete dispersal. Moreover, given the striking effect of MHQ on *P. aeruginosa* adherence, testing the efficacy of the molecule in inhibiting the dynamics of type IV pili of other bacterial species may provide insight into how broadly effective MHQ may be in inhibiting biofilm formation. One of the most surprising aspects of this work is that the authors discovered that conditioned media from the *ΔpqsABCDE* mutant, which does not make detectable levels of MHQ, fully retained dispersal activity. This suggested that, although MHQ was sufficient for surface dispersal, it was not necessary, thereby indicating that an additional dispersal factor or factors secreted by *P. aeruginosa* remain to be discovered! Of course, identification of the additional factor or factors in the conditioned media that also harbor a dispersal activity similar to MHQ may reveal why this process requires separate, redundant mechanisms.

Finally, the study raises an important physiological question about the lifestyle of *P. aeruginosa* that is difficult to address: what is the biological purpose of *P. aeruginosa* harboring this dispersal activity? While the protective benefits of biofilm formation have been self-evident, it remains unclear why *P. aeruginosa* cells may want to coax other *P. aeruginosa* cells to not adhere near them. The authors posit that cells that initially colonize an area and have grown to high density may not welcome cells that are at a different growth stage, for example, and would want to exclude them from joining. Alternatively, it is possible that dispersal represents a response to specific environmental stresses that requires at least part of a population to flee. Clearly, the mechanism must provide a selective advantage to the bacterium, so examining the fitness benefit of cells harboring the dispersal mechanism *versus* those that do not, as well as the cues that induce dispersal activity, may ultimately reveal details of this understudied aspect of the lifestyle of *P. aeruginosa*.

## Conflict of interest

The authors declare that they have no conflicts of interest with the contents of this article.
